# The relationship of autistic traits to taste and olfactory processing in anorexia nervosa

**DOI:** 10.1186/s13229-020-00331-8

**Published:** 2020-04-10

**Authors:** Emma Kinnaird, Catherine Stewart, Kate Tchanturia

**Affiliations:** 1 Department of Psychological Medicine, Institute of Psychology, Psychiatry and Neuroscience, King’s College London, London, UK; 2 Maudsley Centre for Child and Adolescent Eating Disorders, South London and Maudsley NHS Foundation Trust, London, UK; 3 Eating Disorders Service, South London and Maudsley NHS Foundation Trust, London, UK; 4 Department of Psychology, Illia State University, Tbilisi, Georgia

**Keywords:** Anorexia nervosa, Eating disorders, Autism, Sensory, Taste, Olfaction

## Abstract

**Background:**

There is a heightened prevalence of autism in anorexia nervosa (AN) compared to the general population. Autistic people with AN experience a longer illness duration and poorer treatment outcomes. Whether sensory differences in autism could contribute to altered taste and smell as a potential maintaining factor in AN is under-explored. The aim of this study was to explore whether autistic traits are associated with taste and olfaction differences in AN.

**Methods:**

The study recruited *n* = 40 people with AN, and *n* = 40 healthy controls (HC). Smell sensitivity was measured using the Sniffin’ Sticks test. Taste sensitivity was measured using taste strips. Participants self-rated their autistic traits using the Autism Spectrum Quotient.

**Results:**

There were no significant differences on taste and olfactory outcomes between people with AN and HC. These findings did not change after controlling for the heightened levels of autistic traits in the AN group. No relationship between taste and smell outcomes and autistic traits were identified within the AN group.

**Limitations:**

The current study is not able to draw conclusions about taste and smell processing in co-occurring autism and AN as it only measured levels of autistic traits, rather than comparing people with and without an autism diagnosis.

**Conclusions:**

No significant associations between autistic traits and taste and smell processing in AN were identified. Future research should consider further exploring this area, including by comparing autistic women to women with AN.

## Background

Anorexia nervosa (AN) is an eating disorder (ED) characterised by the core symptoms of persistent food restriction, associated low body weight, resistance to weight gain, and body image disturbances [1]. Autistic traits are known to be heightened in AN, with around 20-25% of individuals presenting with clinically significant levels of autistic characteristics on clinical measures [2, 3]. When these measures of current autistic traits are combined with developmental assessments, around 10% of people with AN meet the diagnostic criteria for autism [4]. Significantly, autistic people with AN may experience a longer illness duration, and poorer treatment outcomes in the absence of appropriate adaptations [5–7].

Consequently, there is an increasing interest in exploring characteristics associated with autism that may play a role in maintenance models of AN, contributing to these poorer outcomes. For example, previous studies have investigated similarities in cognitive rigidity and theory of mind difficulties in AN and autism, both of which are thought to act as potential perpetuating factors in the illness [8–10]. To date, there is less research on the implications of sensory sensitivity in autism for AN. Sensory differences driven by alterations in bottom-up processing are common in autism, to the extent that atypical responses to sensory stimuli have been included as a diagnostic criteria in the most recent edition of the Diagnostic and Statistical Manual of Mental Disorders (DSM-5) [1, 11]. Qualitative research on co-occurring autism and AN has highlighted sensory sensitivities as potentially contributing to eating difficulties in AN; however, this area remains under-explored using experimental methods [12, 13]. This is significant as sensory sensitivity may play a role in neurobiological models of AN maintenance. Brain imaging research suggests that food restriction in this condition may be maintained by alterations in neural networks informing appetite regulation, including in areas related to taste and smell processing [14, 15]. Specifically, a number of these studies have detected alterations in the anterior insula in response to taste stimuli [16–18]. The anterior insula is crucial to flavour perception as it integrates taste and olfactory peripheral inputs, although to date no brain imaging studies in AN have examined this area in response to olfactory stimuli [19].

Therefore, alterations in taste and smell sensitivity could potentially contribute to the dysregulation of this neural network, contributing to the key AN symptom of food restriction. Significantly, both taste and olfaction are thought to be altered in autism. Autistic people have a lowered ability to identify tastes, but appear to have normal detection thresholds [20–22]. Systematic reviews suggest that research on olfaction is more mixed [23, 24]. A recent meta-analysis on odour identification and detection thresholds found large heterogeneity in autism, with evidence for both lowered and heightened sensitivity [25].

Whether taste and olfaction are altered in AN remains unclear. As well as responding to the taste and olfactory qualities of food, the anterior insula also appears to be involved in reward and affective assessments [26, 27]. Therefore, from brain imaging evidence only it is difficult to isolate whether alterations in this area suggest altered bottom-up processing of sensory stimuli in AN, or reflect alterations in top-down processing in hedonic and reward related regions [28–30]. Recent systematic reviews on taste and smell in AN highlighted that findings in this area are mixed and inconsistent, ranging from heightened sensitivity, lowered sensitivity, or no significant findings [31, 32]. In the context of these mixed findings, it is possible that taste and smell sensitivity are not consistently altered in AN. Rather, altered sensitivity may be related to specific states associated with AN, rather than representing a core trait of the illness itself. For example, comorbid diagnoses of mood disorders or anxiety are common in this population, and both depression and anxiety are known to be associated with altered taste and olfaction [33–36].

However, to date the potential significance of co-occurring autism in taste and smell sensitivity in AN remains under-explored. To date, two studies on smell sensitivity only have included measures of autistic traits in their analyses with conflicting results; in Bentz et al. (2017), including measures of social and communication differences associated with autism as a covariate in the analysis did not alter their findings of heightend smell sensitivity, and there was no relationship between these differences and sensory outcomes [37]. By contrast, Tonacci et al. (2019) found that adolescents with AN did not differ from healthy controls (HC) on smell sensitivity, but did find a correlation between smell sensitivity and parent-reported autistic traits [38].

Therefore, the aims of this study were to explore whether people with AN experience taste and smell differences compared to HC, and whether taste and smell in AN is associated with autistic traits. In the context of previous mixed findings in the area of sensory sensitivity in AN, this study did not generate specific hypotheses prior to data collection.

## Methods

### Participants

In total, 40 people with AN and 40 HC were recruited into the study. All participants were aged 18-55. Exclusion criteria for all participants included any current medical condition that might affect their taste or smell capacity (for example, neurological disorders such as epilepsy or Parkinson’s disease). Participants experiencing a short-term condition (such as a cold or hay fever) had their testing delayed until after they had recovered.

Participants with AN were recruited from South London and Maudsley NHS Foundation Trust (SLAM) ED service. Additional participants were recruited by advertising online with a UK-based ED charity. Inclusion criteria for people with AN were a diagnosis of AN, confirmed using the Structured Clinical Interview for DSM (SCID-5) [39]. The SCID-5 was additionally used to evaluate whether the participant was experiencing restrictive AN (AN-R) or binge/purge AN (AN-BP).

HCs were included through online advertisements, and through the local university. HCs were excluded if they self-reported a diagnosis of autism, or had ever experienced an ED or other mental health condition. The absence of a previous ED or psychiatric disorder was confirmed using the SCID-5 screening tool prior to testing. The absence of a potential autism spectrum condition was confirmed using the Autism Quotient, with HC only included if they scored below the recommended 32 score threshold [40].

## Measures

### Demographic and clinical information

Participants were asked to self-report information on their age, ethnicity, psychiatric medication use, and whether they currently or previously smoked. People with AN self-reported their illness duration. Individuals with AN currently in treatment had their body mass index (BMI) taken from their most recent measurements in clinical notes. HC and individuals with AN not in treatment had their height and weight assessed on the day of testing.

### Smell sensitivity

The Sniffin’ Sticks extended test (purchased from MediSense) was used to measure three domains of smell sensitivity: odour threshold, odour discrimination, and odour identification [41]. This test was chosen as it is a standardised measure that has been used in the majority of previous research on smell sensitivity in AN [31]. The recommended procedure of the test was followed, with the exception that participants were asked to close their eyes rather than using a blindfold. During the testing, participants are asked to smell felt-tip pens filled with odours, which are held 2 cm in front of the centre of both nostrils. Higher scores on each test indicate higher smell sensitivity.

### Odour threshold

The threshold test assesses at what threshold participants can detect the smell of *n*-butanol across 16 concentrations. Participants close their eyes, and are then presented with three pens in a forced choice paradigm: one pen contains *n*-butanol, whilst the other two contain a non-smelling solvent. Participants are instructed to identify the pen containing the odour. If the participant identifies the pen correctly twice in a row, they are presented with a set of pens at a lower concentration. If they do not correctly identify the *n*-butanol pen, the test proceeds to a set of pens at a higher concentration. The final score is the mean of the last four turning points (where the participant identifies a pen set correctly after previous incorrect identifications, or where the participant incorrectly identifies a pen set after previous correct identifications).

### Odour discrimination

The discrimination test measures the ability to tell the difference between smells. Participants are asked to identify the unique odour pen from a set of 3 pens: each set has 2 pens that smell the same, and one unique pen. This is a forced choice test, and is repeated 16 times with different smell variations. Participants closed their eyes during the test.

### Odour identification

The identification test is a multiple-choice test; the researcher presents participants with one odour pen at a time. Participants then identify the smell from a card with four different options. This is repeated 16 times, with 16 different odours.

### Taste strips

Taste sensitivity was assessed using taste strips, purchased from MediSense [42]. This test was used as it represents a standardised measure of a method widely used in taste research in AN, whereby taste sensitivity is assessed via the administration of tastes at varying concentrations [32]. Taste strips measure taste identification only. Participants are presented with 16 strips of filter paper, each impregnated with four ascending concentrations of the four basic tastes: sweet, salty, sour and bitter. Participants are asked to place the strip in the centre of their tongue, and to identify whether the strip was sweet, salty, sour, bitter or had no taste. Following each strip, participants rinse their mouth with water. Each correct answer yielded one point, giving a maximum score of 16, and 4 for each individual taste quality. Scores were then summed to give a total score, and scores for each taste quality. Higher scores indicate taste smell sensitivity; lower scores indicate lower taste sensitivity.

### Sensory perception quotient

Self-perceived sensitivity to external sensations, such as taste and smell, was measured using the Sensory Perception Quotient (SPQ) [43]. The SPQ was designed to measure sensory perception difficulties associated with autism in adults, and evaluates the five basic exteroceptive sensory modalities (vision, hearing, touch, smell and taste). The participant is presented with 92 different statements on sensory sensitivity, and responds on a four-point Likert scale from ‘strongly agree’ to ‘strongly disagree’. A total of 16 items measure taste, and 16 items measure smell. In contrast to the experimental measures of taste and smell, a higher score indicates lower sensitivity, and a lower score indicates higher sensitivity. This is the first use of the SPQ in people with AN.

### Autism quotient

Participants completed the adult Autism Spectrum Quotient (AQ), a self-report measure designed to screen adults for the presence of autistic traits [40]. Participants respond to 50 statements reflecting autistic traits on a four-point Likert scale from ‘definitely agree’ to ‘definitely disagree’. A higher score indicates higher levels of autistic traits, with a score of ≥ 32 indicating potentially clinically significant levels of autistic traits. The AQ was used in this current study to screen HC for clinically significant levels of autistic traits as it has shown to have good validity distinguishing cases from controls. However, recent studies suggest it may be less effective in predicting an autism diagnosis in clinical populations with high levels of suspected autistic traits [44–46]. In light of this research, the AQ was not used to categorise people with AN in this study as above or below threshold on the AQ, but rather was used as a continuous measure of autistic traits. The AQ has been previously used in AN populations, with people with AN typically scoring higher compared to HC [47].

### Eating disorder examination questionnaire

ED symptoms were measured using the Eating Disorder Examination questionnaire (EDE-Q) [48]. The EDE-Q is a standardised and well validated self-report measure of the severity of the characteristic psychopathology of ED. It contains 36 items which ask respondents to rate how often they have engaged in certain eating disordered behaviours or held disordered concerns over the past 28 days. The scores result in a ‘global’ score that represents the mean of the four subscale scores: ‘eating concern’, ‘weight concern’, ‘shape concern’ and ‘restriction’. Higher scores indicate higher levels of ED symptoms.

### Hospital anxiety and depression scale

The presence of anxiety or depression was assessed using the Hospital Anxiety and Depression Scale (HADS) [49]. The HADS is a widely used 14-item self-rating instrument for anxiety and depression in patients with both physical and mental health problems. The maximum possible score on either subscale (anxiety/depression) is 21, with higher scores indicating higher symptom levels.

### Procedure

All testing took place during one session. Following informed consent, demographic and clinical information was collected from participants. Participants then completed measures in the following order: the self-report questionnaires, the smell tests and finally the taste tests. Participants were given the option to complete the self-report questionnaires before or after the session if preferable. One participant with AN declined to complete the taste test, but participated in the smell test. A small number of participants completed the taste and smell tests, but did not fully complete all self-report questionnaires. Their data has been included in the analysis. Where group sizes vary across measures, this has been highlighted in the result tables.

### Statistical analysis

Data distribution was assessed using Shapiro-Wilk tests and visual checking of histograms. The following variables were found to have a non-normal distribution and so were transformed: age, odour discrimination, and odour identification. However, the following variables could not be transformed and so were analysed using non-parametric tests: BMI, EDE-Q global scores, HADS depression scores, the vision domain of the SPQ and all taste outcomes excepting sour.

Group differences on each variable were initially explored using *t* tests, with Mann-Whitney *U* tests used for non-normal distributions. Effect sizes were calculated using Cohen’s *d*. Categorical variables were compared using Fisher’s exact test. As the analysis involved multiple comparisons, the study used the Bonferroni correction to calculate a more conservative significance value of *p* = 0.005.

Analyses of taste and smell outcomes were then repeated to control for potential confounding variables. Firstly, comparisons were rerun to exclude people who had ever smoked from each group (removing *n* = 10 HC, and *n* = 10 people with AN). Secondly, to control for the potential role of medication use on sensory outcomes, the AN group was split into two sub-groups: (1) those taking psychiatric medication (*n* = 25) and (2) those not taking psychiatric medication (*n* = 15). These sub-groups were then compared on each measure.

After checking for model assumptions, ANCOVAs were performed on the smell outcomes to control for the independent contributions of anxiety and autistic traits in comparisons of sensory variables. An ANCOVA could not be performed on taste outcomes or to control for depression due to data not meeting model assumptions. To explore relationships between sensory outcomes and autistic traits, a multiple regression analysis was run within the AN group only to explore the role of autistic traits whilst controlling for the relative contributions of anxiety and depression. Finally, a Spearman’s rank correlation analysis was performed to assess whether there was any association between age and sensory outcomes.

## Results

### Participant characteristics

Both groups were matched on age, gender, and whether they had ever smoked. Within the AN group, mean illness duration was 9.76 years (*SD* = 8.00). Thirty-four participants (85.00%) reported experiencing AN-R, compared to 6 participants (15.00%) with AN-BP. Eight (20.00%) of participants were in inpatient treatment at the time of the study, 23 (57.50%) participants were attending outpatient treatment, and 9 (22.50%) participants were not in treatment. Twenty-six participants with AN (65.00%) self-reported a diagnosis of at least one comorbid psychiatric condition, including anxiety, depression, obsessive compulsive disorder or borderline personality disorder.

Demographic and clinical characteristics for each group are summarised in Table 1. People with AN had significantly lower BMIs compared to the HC group, and significantly higher levels of ED symptomatology, autistic traits, anxiety and depression.

**Table 1:** Group demographic and clinical characteristics

	HC mean (*SD*) (*n* = 40)	AN mean (*SD*) (*n* = 40)	Test statistic	*p*	Effect size (*d* (95% *CI*))
**Age (years)**	26.45 (7.55)	26.65 (8.60)	*t*(78) = − 0.33	0.741	− 0.07 (− 0.51-0.36)
**Gender**	*n* = 38 female (95.00%) *n* = 2 male (5.00%)	*n* = 38 female (95.00%) *n* = 2 male (5.00%)		1.00	
BMI*	22.5 (4.3)	15.75 (1.22)	*U* = 0	≤ 0.001	2.92 (2.27-3.55)
**Smoking (% never smoked)**	*n* = 30 (75.00%)	*n* = 30 (75.00%)		1.00	
**Psychiatric medication (% currently taking)**	*n* = 0 (0.00%)	*n* = 25 (62.50%)		≤ 0.001	
EDE-Q global*	0.58 (0.9) *n* = 39	4.05 (1.79) *n* = 39	*U* = 74	≤ 0.001	− 2.90 (− 3.54-2.26).
**AQ**	12.48 (6.74)	23.55 (10.26)	*t* (78) = − 5.71	≤ 0.001	− 1.28 (− 1.75-0.79)
HADS depression*	2 (4) *n* = 39	9 (5) *n* = 39)	*U* = 174.5	≤ 0.001	− 1.79 (− 2.32-1.26).
**HADS anxiety**	6.08 (3.80) *n* = 39	13.00 (4.63) *n* = 39	*t* (76) = − 7.21	≤ 0.001	− 1.63 (− 2.14-1.12)

*Indicates non-normally distributed data. Medians and interquartile ranges presented instead of means/standard deviations (*SD*). Effect sizes presented with 95% confidence intervals (*CI*)

### Self-rated sensory sensitivity (SPQ)

**Fig. 1:**
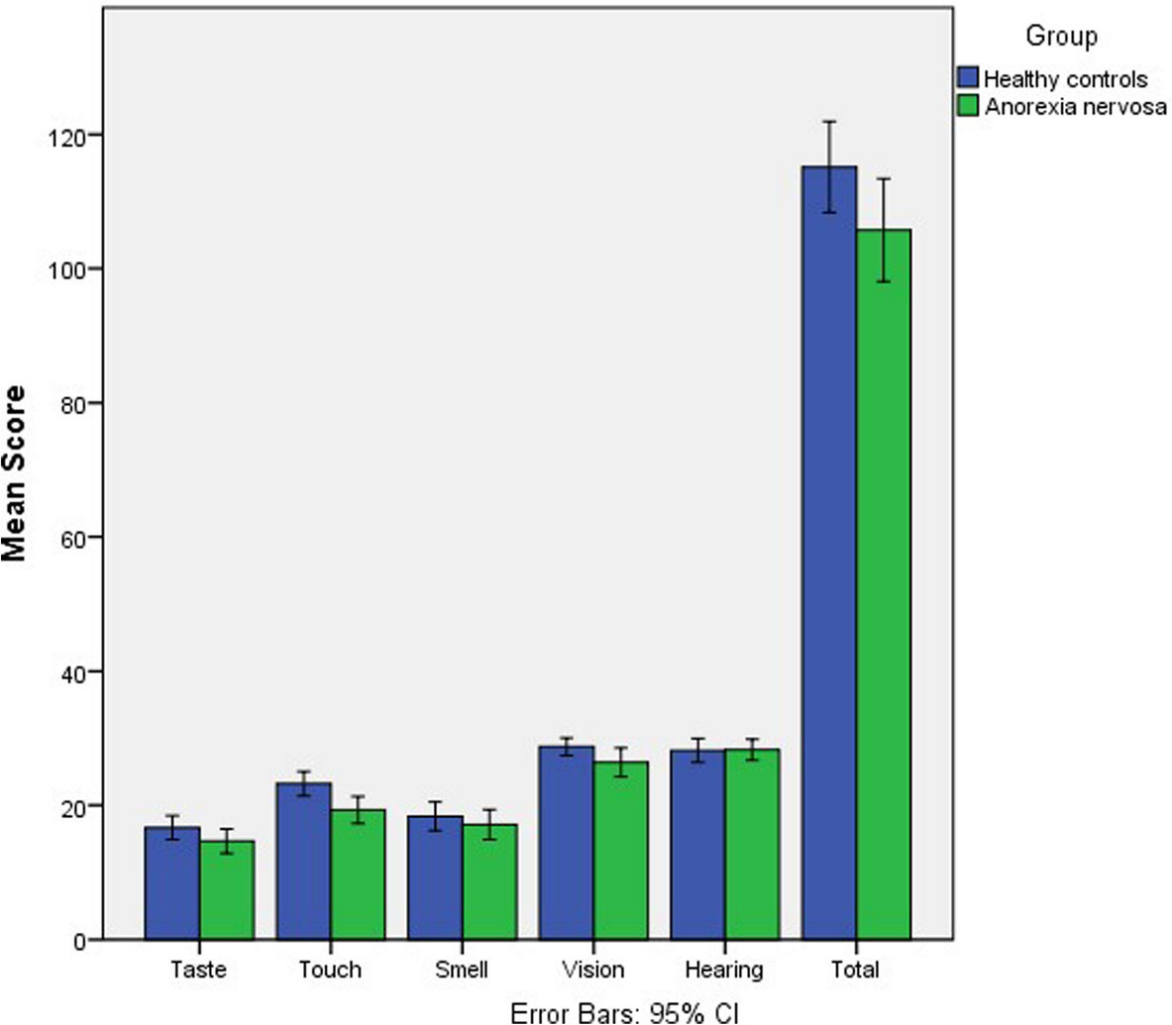
Group scores on the SPQ, summarised by sensory domain and total score

*N* = 38 HC, and *n* = 40 people with AN, completed the SPQ (Fig. 1). People with AN scored significantly lower on touch sensitivity (*t* (76) = 2.90, *p* = 0.005, *d* = 0.66). Lower scores on the SPQ indicate higher self-rated sensitivity, suggesting that people with AN considered themselves to be significantly more sensitive to touch compared to HC. There were no significant differences between AN and HC groups in any other domains.

### Smell and taste sensitivity

Group differences on objective measures of smell and taste sensitivity are summarised in Table 2. There were no significant differences between groups on any outcome measure.

**Table 2:** Taste and smell group outcomes

	HC mean (*SD*) (*n* = 40)	AN mean (*SD*) (*n* = 40)	Test statistic	*p*	Effect size (*d* (95% *CI*))
**Smell total**	36.28 (4.07)	35.7 (4.36)	*t* (78) = 0.62	0.539	0.14 (0.30-0.58)
**Odour threshold**	9.88 (3.09)	10.80 (2.84)	*t* (78) = − 1.38	0.170	− 0.31 (− 0.75-0.13)
**Odour discrimination**	12.98 (1.69)	12.15 (2.35)	*t* (78) = 1.72	0.089	0.39 (− 0.06-0.83)
**Odour identification**	13.43 (1.85)	12.75 (1.69)	*t* (78) = 1.99	0.050	0.45 (0.00-0.89)
	HC mean (*SD*) (*n* = 40)	AN mean (*SD*) (*n* = 39)			
Taste total*	13 (4)	11 (5)	*U* = 628.5	0.135	0.36 (− 0.09-0.80)
Sweet*	4 (1)	4 (1)	*U* = 719	0.502	0.17 (− 0.27-0.61)
**Sour**	2.40 (1.08)	1.82 (1.30)	*t* (77) = 2.16	0.034	0.49 (0.04-0.93).
Salty*	4 (1)	4 (1)	*U* = 742.5	0.686	0.11 (− 0.34-0.55)
Bitter*	3 (2)	3 (2)	*U* = 692.5	0.369	0.21 (− 0.23-0.65)

*Indicates non-normally distributed data. Medians and interquartile ranges presented instead of means/standard deviations (*SD*)

Excluding participants from both groups who ever smoked from the analysis did not change the direction of the results. There were no significant differences on any outcome between participants with AN not taking psychiatric medication, and those currently taking psychiatric medication. Adjusting group comparisons for the heightened levels of anxiety and autistic traits in the AN group using separate ANCOVAs did not alter the results. Result tables for these secondary analyses are located in Additional file 1.

### Relationships between clinical variables

Regression analyses were performed within the AN group to explore the relative contributions of anxiety, depression and autistic traits to sensory measures. With the significance level set at *p* = 0.005, these variables were not found to individually contribute to any sensory outcome at a significant level (Table 3).

**Table 3:** Regression analysis of relative contribution of anxiety, depression and autistic traits to sensory outcomes

	Anxiety (HADS)			Depression (HADS)			Autistic traits (AQ)		
	*Β*	*t*	*p*	*Β*	*t*	*p*	*Β*	*t*	*p*
**Smell total**	0.24	1.35	0.186	− 0.07	− 0.39	0.702	− 0.02	− 0.11	0.913
**Odour threshold**	0.01	0.03	0.975	0.16	0.83	0.414	0.05	0.29	0.772
**Odour discrimination**	0.37	2.13	0.040	− 0.06	− 0.34	0.738	− 0.15	− 0.84	0.407
**Odour identification**	0.10	0.57	0.570	− 0.38	− 2.10	0.043	0.07	0.38	0.706
**Taste total**	0.15	0.83	0.411	− 0.19	− 0.99	0.329	− 0.02	− 0.12	0.906
**Sweet**	0.07	0.40	0.691	− 0.19	− 0.98	0.333	0.02	0.10	0.925
**Sour**	0.32	1.83	0.076	− 0.27	− 1.49	0.145	0.06	0.35	0.726
**Salty**	0.17	0.94	0.354	− 0.14	− 0.76	0.452	− 0.05	− 0.26	0.795
**Bitter**	− 0.11	− 0.58	0.563	0.04	0.20	0.844	− 0.08	− 0.43	0.669

A correlation analysis was also performed to explore whether there was an association between age and sensory outcomes. No significant correlations were found (Table 4).

**Table 4:** Correlation analysis between age and sensory outcomes

	Age (years)	
	*r*	*p*
**Smell total**	0.22	0.171
**Odour threshold**	0.06	0.701
**Odour discrimination**	0.024	0.135
**Odour identification**	0.34	0.031
**Taste total**	0.13	0.419
**Sweet**	− 0.08	0.599
**Sour**	0.11	0.515
**Salty**	− 0.02	0.911
**Bitter**	0.18	0.287

## Discussion

The aims of this study were to explore whether people with AN experience taste and smell differences compared to HC, and whether taste and smell in AN is associated with autistic traits. The analyses identified no significant differences on taste and smell outcomes between people with AN and HC, and no significant associations between taste, smell and autistic traits within the AN group.

Overall, people with AN did not exhibit significant differences on objective measures of sensory sensitivity compared to HC, including after controlling for the potential role of autistic traits. If the absence of significant differences identified in the analysis reflects a true absence of difference, this could suggest that identified alterations in appetite neurocircuitry in AN are driven by differences in top-down processing relating to perceived reward, hedonic and affective salience, rather than bottom-up differences at a sensory level [16–18]. By contrast, previous research in autism has found lowered taste sensitivity [20, 21]. Findings on smell sensitivity in autism are comparably less consistent, with evidence for both heightened and lowered olfaction [25, 50]. If AN and autism are associated with different processes relating to taste, and potentially smell, this could explain why autistic traits were not found to be related to sensory sensitivity in AN in our study.

However, the current study cannot draw conclusions surrounding whether AN and autism are associated with different sensory processing as an autism comparison group was not included. Moreover, previous research on smell and taste processing in autism may lack comparability to studies in AN, including the present paper. The current study, and most previous research on taste and smell sensitivity in AN, has used a predominantly female adult sample [31, 32]. By contrast, research in this area in autism has used predominantly male samples, with a greater focus on children [23–25, 50]. Both age and gender are known to affect sensory processing, particularly in the area of olfaction. Research suggests that olfactory performance improves throughout childhood, reaching adult levels in the later teenage period [51]. Both taste and smell performance then declines after the sixth decade of life [52, 53]. Women also appear to outperform men on smell tests, and there is some evidence that women process tastes differently in the brain [54, 55]. In this context, it is difficult to draw firm conclusions comparing research from these different fields due to the difference in samples. Future research should consider comparing taste and smell processing in autistic people to people with AN, with age and gender-matched samples, to further explore this area.

Previous research on whether there is a relationship between smell sensitivity and autistic traits in AN has yielded mixed results (to date, no study has examined taste sensitivity). One study found a relationship between parent-reported autistic traits and olfaction [38], and another found no relationship between social and communicative characteristics of autism and smell outcomes [37]. It should be noted that autistic traits have not consistently found to be related to smell sensitivity even in autistic populations, with studies finding conflicting results [50, 56, 57]. One possibility for these mixed findings is that the heightened levels of autistic traits influence smell outcomes via a secondary, mediating variable. For example, anxiety and sensory sensitivity are known to be interrelated features in autism [58]. A strength of the present study is that the relationship of autistic traits and sensory outcomes in AN was analysed relative to the contributions of anxiety and depression using a multiple regression analysis. This is important as both anxiety and depression are known to influence taste and smell processing, including in AN [33, 34, 37]. The lifetime prevalence rate of anxiety in autistic adults is 42%, and 37% for depression [59]. Additionally, anxiety has been shown to correlate with autistic traits in AN [60]. Therefore, the finding of this study that autistic traits were not significantly related to objective measures of taste and smell sensitivity are likely to be more robust against the influence of these interrelated variables. However, the current study did not explore IQ as a potential confounding variable, although it has previously been identified as a moderating factor in smell sensitivity in autism [25]. Additionally, this study did not control for age beyond excluding people aged over 55 years, although no significant relationship between age and sensory outcomes was identified in the current study. Therefore, future research exploring smell sensitivity, autism and anorexia could further consider the potential role of mediating variables in this relationship.

On self-report measures people with AN exhibited no significant differences in taste and smell sensitivity compared to HC. Previous research indicates that people with AN may rate themselves as significantly more sensitive to sensations, and may be more likely to attempt to avoid sensory stimuli compared to HC [61, 62]. As the self-report measures in this study reflect the objective outcomes, it is possible that the SPQ represents an accurate measure of sensory sensitivity only in this population, whereas previous studies additionally measured affective or hedonic responses to sensory stimuli [61, 62]. In the current study, people with AN only rated themselves as more sensitive to touch. The area of touch sensitivity has primarily been explored through the paradigm of affective touch: people with AN perceive affective, interpersonal touching as less pleasurable compared to HC [63]. Future research could consider if the pleasurable aspects of interpersonal touch may also be related to an underlying sensory hypersensitivity to touch itself.

## Limitations

A limitation of this study was that it measured self-rated levels of autistic traits in people with AN, rather than comparing autistic people with AN to those with AN only. Therefore, it cannot draw conclusions on whether these two groups do experience differences in taste and smell sensitivity. Further research should consider autistic people to people with AN only, including matching for age and gender. Future research could also explore using clinical and developmental autism assessments to establish a co-occurring autism/AN group, and compare to people with AN only and HC. An additional limitation of this study is that whilst it used a heterogenous AN sample that may better reflect clinical variety, the sample was not large enough to control within the analysis for potential confounding factors introduced by this heterogeneity. For example, the current study did not explore the potential role of illness duration, BMI or comorbidities beyond anxiety and depression.

Furthermore, although the findings of this study were non-significant, the methods used cannot account for whether this reflects a true absence of difference between groups, or whether these non-significant findings result from chance or a lack of power [64]. However, sample size calculations to determine the minimum sample size required for adequate study power were calculated a priori to recruitment on the basis of two previous studies in AN which measured the same taste and smell outcomes as the present research, and which reported means and standard deviations [65, 66]. These calculations suggested that a minimum sample size approximating *n* = 20 in each group would give sufficient statistical power, although the current study recruited larger groups due to its aim to investigate potential covariates. This indicates that the lack of significant findings is unlikely to be due to a lack of power. Additionally, the finding that confidence intervals for the majority of taste and smell outcomes includes 0 supports an absence of significant difference. However, without a Bayesian approach to analysis the current study cannot precisely quantify the level of evidence for whether there is truly no difference between groups.

## Conclusions

The study found that taste and smell sensitivity was not significantly altered in AN, with no significant relationship between these outcomes and autistic traits within the AN group. In the context of previous studies suggesting altered taste and smell sensitivity in autism, future research should explore sensory differences in autistic people compared to people with AN, and implications for individuals with co-occurring autism and AN.

## Supplementary information

**Additional file 1.** Secondary Analysis Results. File including results tables for secondary analyses.

## Data Availability

The datasets used and/or analysed during the current study are available from the corresponding author on reasonable request.

## Abbreviations

AN: Anorexia nervosa
AN-BP: Anorexia nervosa, binge-purge subtype
AN-R: Anorexia nervosa, restrictive subtype
AQ: Autism Quotient (Adult)
BMI: Body mass index
CI: Confidence intervals
DSM: Diagnostic and Statistical Manual of Mental Disorders
ED: Eating disorder
EDE-Q: Eating Disorder Examination Questionnaire
HADS: Hospital Anxiety and Depression Scale
HC: Healthy control
SCID-5: Structured Clinical Interview for DSM
SD: Standard deviations
SLAM: South London and Maudsley NHS Foundation Trust
SPQ: Sensory Perception Quotient
UK: United Kingdom
