# Supplementary material for: The relationship of autistic traits to taste and olfactory processing in anorexia nervosa
Source: Mol Autism. 2020 Apr 10;11:25. doi: 10.1186/s13229-020-00331-8 (PMC7146886; doi:10.1186/s13229-020-00331-8)
Supplement: Supplementary file 1 — Additional file 1. Secondary Analysis Results. File including results tables for secondary analyses. [file 13229_2020_331_MOESM1_ESM.docx]

**Additional File 1: Secondary Analysis Results.**

This file includes the results tables for secondary analyses run to control for potential confounders following initial group comparisons.

**Analysis 1: Excluding people who had ever smoked from the analysis.**

Comparisons were rerun to exclude people who had ever smoked from each group (removing *n*= 10 HC, and *n*= 10 people with AN). This did not alter the direction of results (Table 1).

Table 1: Taste and smell group outcomes, adjusted for smoking status.

|  | **HC mean (*SD*) (*n*=30)** | **AN mean (*SD*) (*n*=30)** | **Test statistic** | ***p*** |
| --- | --- | --- | --- | --- |
| **Odour Total** | 36.28 (3.90) | 35.33 (4.13) | *t*(58)= 0.92 | 0.364 |
| **Odour Threshold** | 10.22 (2.74) | 10.87 (2.77) | *t*(58)= -0.92 | 0.364 |
| **Odour Discrimination** | 12.67 (1.60) | 11.87 (1.52) | *t*(58)= 1.27 | 0.208 |
| **Odour Identification** | 13.40 (2.01) | 12.60 (1.59) | *t*(58)= 2.17 | 0.034 |
|  | **HC mean (*SD*) (*n*=30)** | **AN mean (*SD*) (*n*=29)** |  |  |
| **Taste Total** | 13(5) | 11(5) | *U*= 335.5 | 0.129 |
| **Sweet*** | 3.5 (1.5) | 4 (1) | *U*= 425.5 | 0.874 |
| **Sour** | 2.33 (1.21) | 1.66 (1.29) | *t*(57)= 2.08 | 0.042 |
| **Salty*** | 4 (1) | 3 (1) | *U*=384.5 | 0.407 |
| **Bitter*** | 3 (1) | 3 (2) | *U*= 346.5 | 0.161 |

**Analysis 2: Comparing people with AN taking psychiatric medication, and those not taking medication.**

To control for the potential role of medication use on sensory outcomes, the AN group was split into two sub-groups: 1) those taking psychiatric medication (*n*= 25) and 2) those not taking psychiatric medication (*n*= 15). These sub-groups were then compared on each measure (Table 2).

Table 2: Taste and smell group outcomes, comparing people with AN based on psychiatric medication use.

|  | **AN medication mean (*SD*) (*n*=25)** | **AN no medication mean (*SD*) (*n*=15)** | **Test statistic** | ***p*** |
| --- | --- | --- | --- | --- |
| **Odour Total** | 35.94 (4.23) | 35.30 (4.68) | *t*(38)= -0.45 | 0.659 |
| **Odour Threshold** | 10.66 (2.75) | 11.03 (3.06) | *t*(38)= 0.40 | 0.693 |
| **Odour Discrimination** | 12.40 (1.78) | 11.73 (3.10) | *t*(38)= -0.47 | 0.641 |
| **Odour Identification** | 12.88 (1.54) | 12.53 (1.96) | *t*(38)= -0.49 | 0.625 |
|  | **AN medication mean (*SD*) (*n*=24)** | **AN no medication mean (*SD*) (*n*=15)** |  |  |
| **Taste Total*** | 11.5 (4) | 11 (6) | *U*= 172 | 0.816 |
| **Sweet*** | 4 (1) | 4 (1) | *U*= 174 | 0.848 |
| **Sour** | 1.58 (1.47) | 2.2 (0.86) | *t*(37)= 1.47 | 0.150 |
| **Salty*** | 4 (1) | 3 (1) | *U*= 160.5 | 0.540 |
| **Bitter*** | 3 (2) | 3 (1) | *U*= 151 | 0.385 |

**Analysis 3: ANCOVA to control for anxiety.**

An ANCOVA was performed on the smell outcomes to control for the independent contributions of anxiety to group comparisons (Table 3).

Table 3: Smell outcomes adjusted for heightened levels of anxiety in the AN group.

|  | **Adjusted HC mean (*95% CI*)** | **Adjusted AN mean (*95% CI*)** | **Test statistic** | ***p*** |
| --- | --- | --- | --- | --- |
| **Odour Total** | 36.91 (35.38- 38.45) | 34.87 (33.34- 36.41) | *F*(1, 78)= 2.78 | 0.099 |
| **Odour Threshold** | 10.09 (8.99- 11.20) | 10.52 (9.41- 11.62) | *F*(1, 78)= 2.10 | 0.630 |
| **Odour Discrimination** | 13.44 (12.70- 14.18) | 11.72 (10.98- 12.46) | *F*(1, 78)= 6.32 | 0.014 |
| **Odour Identification** | 13.39 (12.74- 14.03) | 12.64 (12.00-13.28) | *F*(1, 78)= 2.11 | 0.151 |

**Analysis 4: ANCOVA to control for autistic traits.**

An ANCOVA was performed on the smell outcomes to control for the independent contributions of autistic traits to group comparisons (Table 4).

Table 4: Smell outcomes adjusted for heightened levels of autistic traits in the AN group.

|  | **Adjusted HC mean (*95% CI*)** | **Adjusted AN mean (*95% CI*)** | **Test statistic** | ***p*** |
| --- | --- | --- | --- | --- |
| **Odour Total** | 36.00 (34.54- 37.46) | 35.98 (34.52- 37.44) | *F*(1, 80)= 0.00 | 0.984 |
| **Odour Threshold** | 9.93 (8.90- 10.97) | 10.75 (9.72- 11.78) | *F*(1, 80)= 1.06 | 0.307 |
| **Odour Discrimination** | 12.86 (12.15-13.57) | 12.27 (11.56-12.98) | *F*(1, 80)= 0.76 | 0.387 |
| **Odour Identification** | 13.21 (12.61-13.82) | 12.96 (12.36-13.57) | *F*(1, 80)= 0.48 | 0.491 |
